# A Comparison of the bébé VieScope™ and Direct Laryngoscope for Use While Wearing PPE-AGP: A Randomized Crossover Simulation Trial

**DOI:** 10.3390/children9111774

**Published:** 2022-11-18

**Authors:** Pawel Wieczorek, Lukasz Szarpak, Agata Dabrowska, Michal Pruc, Alla Navolokina, Andrzej Raczynski, Jacek Smereka

**Affiliations:** 1 Pediatric Intensive Care Unit (PICU), John Paul II Upper Silesian Health Centre in Katowice, 40-752 Katowice, Poland; 2 Research Unit, Polish Society of Disaster Medicine, 05-806 Warsaw, Poland; 3 Research Unit, Maria Sklodowska-Curie Bialystok Oncology Center, 15-027 Bialystok, Poland; 4 Henry JN Taub Department of Emergency Medicine, Baylor College of Medicine Houston, Houston, TX 77030, USA; 5 Department of Rescue and Disaster Medicine, Poznan University of Medical Sciences, 60-806 Poznan, Poland; 6 European School of Medicine, International European University, 03187 Kyiv, Ukraine; 7 Department of Emergency Medical Service, Wroclaw Medical University, 52-443 Wroclaw, Poland

**Keywords:** endotracheal intubation, pediatric, direct laryngoscopy, VieScope, Macintosh laryngoscope, FPS, success rate, intubation time, medical simulation

## Abstract

This study aimed to compare the intubation effectiveness of the bébé Vie Scope™ (VieScope) and direct laryngoscopy for emergency intubation in a pediatric manikin model performed by paramedics with and without personal protective equipment for aerosol generating procedures (PPE-AGP). Participants performed endotracheal intubation using VieScope and standard Macintosh laryngoscope (MAC) in two research scenarios: (1) without PPE-AGP, and (2) with PPE-AGP. Fifty-one paramedics without any previous experience with the VieScope participated in this study. In the PPE-AGP scenario, in the VieScope group, the percentage of successful tracheal intubation on the first attempt was higher compared to the MAC group (94.1 vs. 78.4%, *p* = 0.031), intubation time was shorter (29.8 vs. 33.9 s, *p* < 0.001), and percentage of glottic opening (POGO) score was higher 91.0 vs 77.8 (*p* < 0.001). On the Cormack–Lehane scale, intubation with VieScope intubation was associated with higher scores rated at 1 (64.7 vs. 29.4%) than in the MAC group (*p* = 0.001). For intubation in the non-PPE scenario, there were no statistically significant differences between VieScope and MAC in relation to above parameters. Summarize, the bébé VieScope™ under PPE-AGP wearing conditions has proven to be a useful device for airway management in children providing better visualization of the larynx, better intubation conditions, and a higher success rate of tracheal intubation on the first attempt and reduced intubation time compared to the standard Macintosh laryngoscope.

## 1. Introduction

The COVID-19 pandemic poses a challenge in many respects, including medical personnel safety. Performing aerosol-generating procedures (AGP) is associated with the risk of medical personnel infection, necessitating the use of full personal protective equipment (PPE) [1,2]. Performing some of the medical procedures in PPE may involve technical difficulties [3], which in emergency medicine may also apply to advanced airway management, including endotracheal intubation [4]. 

Emergency endotracheal intubation in children is often performed by medical personnel, who are much more experienced in intubating adults than children. Emergency endotracheal intubation in children can be associated with a significant risk of failure, which can be compounded by the emergency associated with the aerosol-generating procedure and the need to apply PPE [5]. Complications of improperly performed endotracheal intubation can include inadequate ventilation, severe cerebral hypoxia leading to death, dental injury, and soft tissue injury to the upper airway [6]. Repeated unsuccessful attempts at endotracheal intubation can lead to hypoxia and death of the child, which is particularly important in children with severe respiratory failure and a lack of respiratory reserve in the case of COVID-19.

The main issue is the fact that the different anatomical anatomy of the upper airway and the elements that affect the difficulty of airway management in children and the significant difference in the experience of endotracheal intubation by medical personnel of adults and children in real clinical conditions.

Endotracheal intubation is routinely performed with direct laryngoscopy. However, due to the problems associated with emergency endotracheal intubation in pediatric patients, there are many studies showing the effectiveness of indirect laryngoscopy using video laryngoscopy [7]. The use of video laryngoscopy in pediatric patients is associated with better visualization of the entrance to the larynx, but there is ongoing debate as to whether this converts into an increase in the percentage of successful intubations at first attempt and a shortening of the procedure itself [8,9,10].

**Figure 1:**
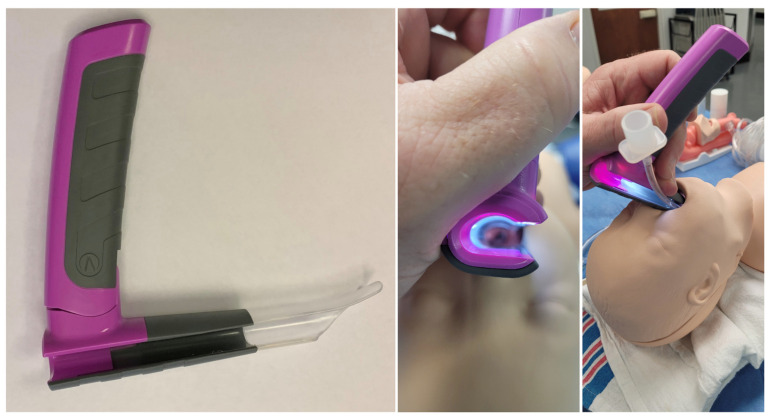
The bébé Vie Scope™ (Android Surgical^TM^, Oklahoma, OK, USA).

The bébé Vie Scope™ (VieScope) is based on the Parsons laryngoscope, a commonly used ENT device (Figure 1). Unlike the many sizes available with the Parsons, the VieScope design has been modified so one size can be used for toddlers to children up to 10 years of age, depending on the size. 

The VieScope has a larger right-sided open slot along its entire length to allow the introduction of an age-appropriate endotracheal tube in addition to a bougie. The bébé VieScope shares the same proximal illumination system as the Vie Scope to avoid blackouts from blood and secretions. The blade tip is versatile as it can be used like a Macintosh with the blade in the vallecular and as a Miller blade to elevate the epiglottis, depending on the child’s anatomy. A larger and longer tooth guard protects the gums and teeth.

Therefore, this study aimed to compare the first-attempt intubation success rate of the VieScope and direct laryngoscopy for emergency intubation in a pediatric manikin model performed by paramedics with and without PPE-AGP.

## 2. Materials and Methods

This study was designed as a prospective, observational, randomized, crossover simulation trial. Before the commencement of the study, the study protocol was approved by the Institutional Review Board of the Polish Society of Disaster Medicine (Approval No. 22.07.2022.IRB). The study is a continuation of the research conducted by the authors on the determination of the most effective method of endotracheal intubation for patients in a state of life-threatening conditions [11,12].

Paramedics working as part of the National Emergency Medical Service were recruited during training courses conducted as part of the “Introduction to tactical medicine” courses. Participants were recruited between June and October 2022, and all participants provided voluntary informed consent. All study participants had at least two years of experience in prehospital emergency settings. Paramedics without a minimum of two years of professional experience were excluded from the study. Study participants who had any previous experience or received training in the use of the VieScope laryngoscope for adults or children were excluded from the study.

### 2.1. Scenario Design

Two laryngoscopes were compared in the study:

(a) The bébé VieScope™ laryngoscope (VieScope; Adroit Surgical LLC, Oklahoma, OK, USA).
(b) Macintosh laryngoscope (MAC) with the blade No. 2 (HEINE Optotechnik GmbH & Co. KG, Filching, Deutschland). This type of laryngoscope, due to its prevalence both in prehospital care and in Emergency Departments, has been recognized as the “gold standard”.

Before starting the study, all participants took part in a 60-min training on airway management in pediatric patients suspected of having an infectious disease (COVID-19). During the training, the instructor demonstrated the correctness of endotracheal intubation using VieScope and MAC. Subsequent study participants had the option of a 10-min training session with each device under normal airway conditions. For this purpose, the Pediatric Intubation Skill Trainer (Laerdal, Stavanger, Norway) was used, which was designed to map the respiratory tract of a 6-year-old child.

To simulate a pediatric patient with suspected COVID-19, the HAL^®^ S3005 Five-Year-Old Advanced Pediatric Patient Simulator (Gaumard, Miami, FL, USA) was used. The simulator was placed on the floor level in a neutral position. Then, during the target study, participants were asked to perform endotracheal intubation using the tested laryngoscopes in two research scenarios: (1) without PPE-AGP (Scenario A) and (2) with PPE-AGP (Scenario B).

To ensure the condition of wearing PPE-AGP, the Tychem F chemical-resistant suit was used, the characteristics of which protect against high concentrations of organic and inorganic chemical particles and those with a diameter of fewer than one μm (DuPont Personal Protection, Luxembourg). The subjects’ airways were protected using a protective mask equipped with an FFP2 filter (3M Aura Disposable Respirator, FFP2, Valved, 9312+, 3M Inc., Bracknell, UK), the eyeballs were protected with protective googles (MedaSEPT, Poznan, Poland) and visors, and double nitrile gloves were also used for hand protection. 

**Figure 2:**
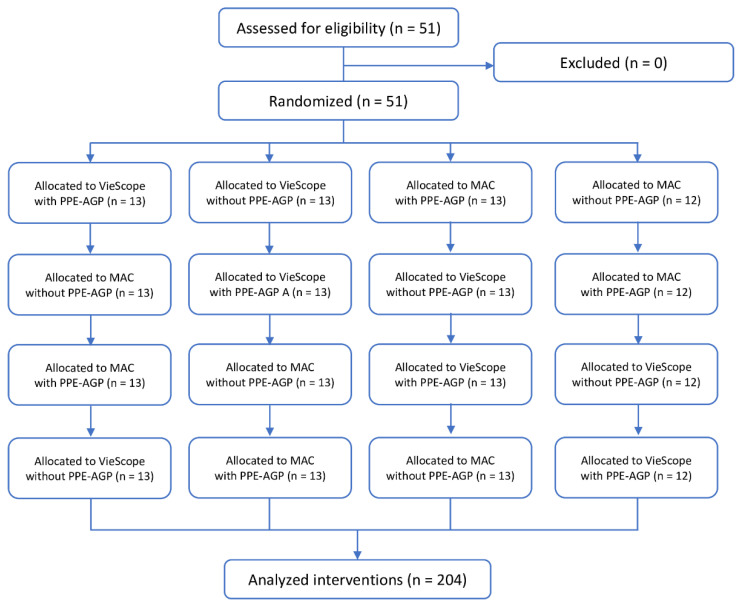
Randomization flow chart. *Legend: MAC = Macintosh laryngoscope; PPE-AGP = personal protective equipment for aerosol-generating procedures*.

Both the order of participants and intubation methods were randomized. For this purpose, the ResearchRandomizer program was used [13] (Figure 2). In each scenario, participants had only one attempt to intubate with each laryngoscope.

### 2.2. Data Collection

The primary outcome was the first pass success rate (FPS). FPS was recorded when the simulator’s ventilation indicators confirmed the success of the ventilation attempt. Secondary outcomes include time to intubate, glottis visualization based on Cormack–Lehane classification [14], and percentage of glottis opening (POGO) score. Time to intubation was defined as starting when the blade of the laryngoscope was inserted between the teeth and timing finished at the first ventilation of the lung. Each time was measured using a stopwatch by the same instructor. 

### 2.3. Statistical Analysis

For this study, the sample size was based on G*Power 3.1 using a 2-tailed *t*-test. A minimum of 39 paramedics were necessary to achieve a Cohen d = 0.8. The sample size was calculated with G*Power 3.1 using a 2-tailed *t*-test. A minimum of 39 paramedics were necessary to achieve a Cohen d = 0.8, alpha error = 0.05, and power = 0.95). To provide a safety margin in case of missing data or non-participation, we increased the minimum size of the study group to 51 participants.

Data were recorded in an Excel worksheet and the statistical analysis was performed using Statistica software version 13.4 EN (Tibco Inc., Tulsa, OK, USA). Depending on the type of data, they are presented as mean (standard deviation (SD)) or numbers (with percentages shown) as appropriate. In the absence of normal distribution, non-parametric tests were used, including the Shapiro–Wilk test and Lilliefors test. A one-way ANOVA on ranks was used to analyze procedure times and compare these values between groups, with a post hoc Bonferroni correction to counteract the multiple comparisons. Two-sided statistical tests were performed in all relevant cases. The analysis accepted *p* < 0.05 as statistically significant.

## 3. Results

The results show the data obtained for endotracheal intubation performed with a VieScope and MacIntosh laryngoscope (MAC) when using PPE (with PPE-AGP) and without PPE (without PPE-AGP).

### 3.1. Intubation without PPE-AGP Scenario

**Figure 3:**
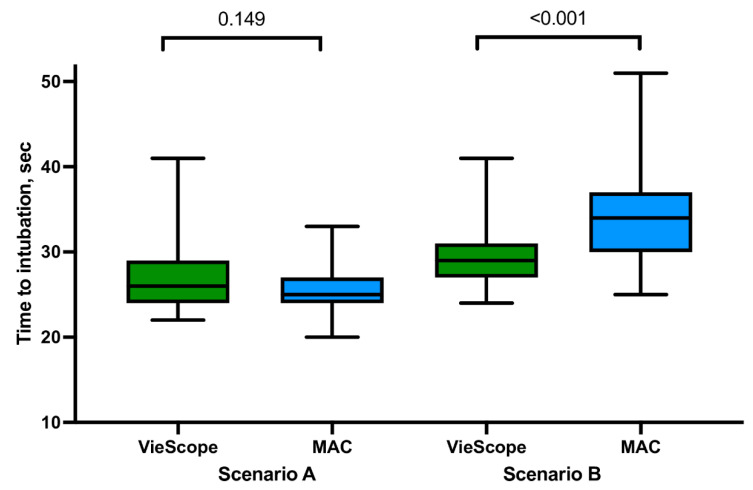
Time to intubation among evaluated intubation methods. *Legend: MAC = Macintosh laryngoscope; Scenario A = intubation without personal protective equipment for aerosol-generating procedures (PPE-AGP); Scenario B = intubation with PPE-AGP*.

**Figure 4:**
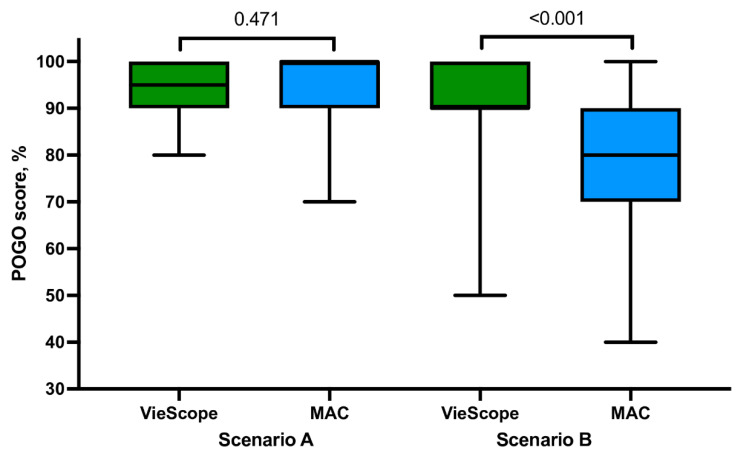
Percentage of glottis opening score among evaluated intubation methods. *Legend: MAC = Macintosh laryngoscope; Scenario A = intubation without personal protective equipment for aerosol-generating procedures (PPE-AGP); Scenario B = intubation with PPE-AGP*.

For intubation in the non-PPE scenario, there were no statistically significant differences between VieScope and MAC in the percentage of successful tracheal intubation on the first attempt (98.0 vs. 92.2%), median intubation time (27.0 vs. 25.9 s; Figure 3), POGO score (95.4 vs. 94.2), and Cormack–Lehane scale score (Figure 4). In addition, study participants rated, on the ease of intubation scale, that intubation with MAC was easier (23.9 vs. 22.2, *p* = 0.002). Detailed information is presented in Table 1.

### 3.2. Intubation with PPE-AGP Scenario

In the PPE-AGP scenario, statistically, significantly different results were found for all measured parameters. In the VieScope group, the percentage of successful tracheal intubation on the first attempt was higher compared to the MAC group (94.1 vs. 78.4%, *p* = 0.031), intubation time was shorter (29.8 vs. 33.9 s, *p* < 0.001), and POGO score was higher 91.0 vs. 77.8 (*p* < 0.001). On the Cormack–Lehane scale, intubation with VieScope intubation was associated with higher scores rated at 1 (64.7 vs. 29.4%) than in the MAC group (*p* = 0.001). Study participants rated, on the ease of intubation scale, that intubation with MAC was easier (40.8 vs. 27.5, *p* ≤ 0.001). Detailed information is presented in Table 2.

### 3.3. Influence of PPE-AGP in Intubation with VieScope Laryngoscope

When comparing intubation using VieScope according to PPE-AGP, there were non-statistical differences in the percentage of successful tracheal intubation on the first attempt (*p* = 0.159) and Cormack–Lehane grade (*p* = 0.371). However, in non-PPE-AGP settings, intubation time was shorter (27.0 vs. 29.8 s, *p* < 0.001), and the POGO score was higher, 95.4 vs. 91.0 (*p* = 0.006), compared to PPE-AGP settings. 

### 3.4. Influence of PPE-AGP in Intubation with Macintosh Laryngoscope

When comparing intubation using MAC according to PPE-AGP, statistically significantly different results were found for all measured parameters. In the PPE-AGP group, the percentage of successful tracheal intubation on the first attempt was lower (78.4 vs. 92.2%, *p* = 0.070), intubation time was longer (33.9 vs. 25.9 s, *p* < 0.001), POGO score was lower (77.8 vs. 94.2, *p* < 0.001) compared to the non-PPE-AGP group. Study participants rated, on the ease of intubation scale, that intubation without PPE-AGP was easier (40.8 vs. 23.9, *p* < 0.001). On the Cormack–Lehane scale, intubation with MAC laryngoscope without PPE-AGP was associated with higher scores rated at 1 (76.5 vs. 29.4) than in the PPE-AGP group (*p* = 0.001). 

## 4. Discussion

The present study demonstrates the advantages and disadvantages of using VieScope in the pediatric patient population, mainly when it is necessary to operate in an environment exposed to the formation of a highly infectious aerosol. It can be assumed that the general principles of securing medical personnel and the need to work and perform complex medical procedures in PPE will be related to the current COVID-19 pandemic and potentially to other pathogens, infections, and threats we may encounter in the future [2]. 

Airway management, when performed in an environment with a high risk of contagious infectious diseases, including the SARS-CoV-2 pandemic, is an important issue due to the potential difficulties and the need to specifically avoid cross-contaminations of contagious equipment and material [4,15]. Of particular importance is airway management in pediatric patients due to the less experience of medical personnel in these activities in the pediatric group and the potential problem of airway management in children. Taking into account both of these factors—the risk to the rescuer, the need for full personal protective equipment and specific airway management in children—in a population where most of those involved in airway management have less experience, this situation may generate additional risks and requires special evaluation and attention.

The VieScope is a relatively new device for airway management. A pediatric version of the VieScope device, the bébé VieScope™, has recently become available and approved for clinical use. To the best of the authors’ knowledge, no study has been published on the use of the bébé VieScope™, in the pediatric patient population, either in real-life medical conditions or with the use of medical simulation. 

The VieScope device has been evaluated on adult populations in several studies using medical simulation and clinical trials. Due to the lack of other studies and the novel nature of the results, it is difficult to directly reference other studies on pediatric patients. Some authors have evaluated the feasibility of using a general version of VieScope, not specifically designed for pediatric patients, for airway management in children in a medical simulation setting.

Maslanka et al. examined the suitability of VieScope for endotracheal intubation on a pediatric model in a randomized, cross-over simulation study [11]. The authors found on a group of 55 paramedics in each of the scenarios tested—normal airways, tongue edema, and cardiopulmonary resuscitation—that the overall intubation success rate was 100% and the median intubation time was 27–29 s depending on the scenario; however, they use the adult version of VieScope in their study. In our study using the VieScope™ bébé in normal airways, we obtained similar results (27–30 s) dependent on the use of PPE-AGP by the participants. This may indicate the high reproducibility of the results obtained and the suitability of the bébé VieScope™ for intubation on a pediatric patient model. The manufacturer’s development of a pediatric version of the VieScope laryngoscope may create further opportunities to use the device on a group of pediatric patients.

However, most of the available studies related to using standard VieScope in adults and the device have been tested in both medical simulation and clinical settings. In a randomized clinical trial presented by Sharpak et al., the authors presented the use of VieScope in the intubation of adults in prehospital settings during the COVID-19 pandemic [16]. The paramedics used Vie Scope^®^ or classic direct laryngoscopy with a Macintosh blade for airway management in adults in sudden cardiac arrest and were wearing personal protective equipment. The authors evaluated the success of intubation attempts, time to intubation, glottis visualization, and the number of optimization maneuvers in the study and control groups. In a group of 90 out-of-hospital cardiac arrest patients aged 43-92 years, they found that VieScope^®^ provided a first-attempt success rate in 93.3% of cases, while in the Macintosh laryngoscope group, only 51.1%. Intubation with VieScope^®^ took less time compared to Macintosh laryngoscope in paramedics wearing PPE for aerosol-generating procedures. 

Petzoldt et al. published a study on elective endotracheal intubation using VieScope and Macintosh laryngoscope in adult patients [17]. They measured first-attempt success rates, percentage of glottis opening scale, time to intubation, and difficulty ratings on visual analogue scales. This study showed no significant advantage of VieScope over classic intubation using a MacIntosh laryngoscope. The first-attempt intubation success rate was comparable in VieScope and Macintosh laryngoscope groups, but the time to intubation with VieScope was statistically significantly longer. They concluded that visualization of the larynx was superior using the VieScope, and it can be an alternative to Macintosh laryngoscopy in patients with a difficult airway.

Ecker et al. published a study regarding the use of VieScope in the adult population in a randomized, controlled simulation study evaluating time to intubation and the intubation success and extension of pulmonary on an airway manikin simulating massive regurgitation of gastric fluid [18]. It has been shown that laryngoscopy using Macintosh can provide faster airway management with less gastric aspiration compared to VieScope. This study was conducted on an adult model with specific conditions of massive regurgitation of gastric fluid.

Ecker et al. also compared VieScope Glidescope, Glidescope, and conventional Macintosh laryngoscopy in a simulated randomized controlled simulation trial in a difficult airway situation [18]. Those authors concluded that the correct tracheal tube position rate was comparable between the three devices. Still, the time for intubation and ventilation was shorter with Macintosh and Glidescope compared to VieScope. In their study, the mean intubation time for VieScope was 36.3 s. However, this study was conducted on an adult model with a difficult airway, and anesthesiologists carried out the intubation with substantial experience in endotracheal intubation. 

In a randomized, controlled simulation trial, Ecker et al. also compared VieScope to video laryngoscopy in full personal protective equipment [15]. This study revealed that VieScope and GlideScope had high success rates in normal and difficult airways. Results from this study suggested that VieScope may be an acceptable alternative for tracheal intubation in full PPE.

The study we conducted has several limitations. The study is a pilot study on a newly introduced device specifically designed for a group of pediatric patients. The results obtained cannot be directly transferred to clinical conditions due to the simulation nature of the study. An important factor is also the issue of the experience that individual study participants have in airway management. The study was limited to paramedics and cannot be directly transferred to physicians and other medical personnel with different experiences in airway management. The study did not evaluate complications associated with intubation by selected methods, including using VieScope and the bébé VieScope. The anatomical conditions reflected by the phantom may differ from actual intubation conditions in different age groups of pediatric patients.

## 5. Conclusions

The bébé VieScope™ under PPE-AGP wearing conditions in a randomized crossover simulation trial has proven to be a useful device for airway management in children providing better visualization of the larynx, better intubation conditions and a higher success rate of tracheal intubation on the first attempt and reduced intubation time compared to standard MacIntosh laryngoscope.

## Figures and Tables

**Table 1:** Intubation without personal protective equipment for aerosol-generating procedures (PPE-AGP) scenario.

Parameter	VieScope	MAC	*p*-Value
FPS, n (%)	50 (98.0%)	47 (92.2%)	0.182
Time to intubate (s), mean (SD)	27.0 (4.1)	25.9 (2.8)	0.149
Cormack–Lehane grade			
1	35 (68.6%)	39 (76.5%)	0.419
2	16 (31.4%)	12 (23.5%)	
3	0 (0.0%)	0 (0.0%)	
4	0 (0.0%)	0 (0.0%)	
POGO score, mean (SD)	95.4 (5.0)	94.2 (9.1)	0.471
Ease of intubation (1–10), mean (SD)	22.2 (7.6)	23.9 (6.7)	0.002

Legend: MAC = Macintosh laryngoscope; FPS = first pass success rate; SD = standard deviation; POGO = percentage of glottis opening.

**Table 2:** Intubation with personal protective equipment for aerosol-generating procedures (PPE-AGP) scenario.

Parameter	VieScope	MAC	*p*-Value
FPS, n (%)	48 (94.1%)	40 (78.4%)	0.031
Time to intubate (s), mean (SD)	29.8 (3.6)	33.9 (5.4)	<0.001
Cormack–Lehane grade			
1	33 (64.7%)	15 (29.4%)	0.001
2	17 (33.3%)	34 (64.8%)	
3	1 (2.0%)	2 (5.8%)	
4	0 (0.0%)	0 (0.0%)	
POGO score, mean (SD)	91.0 (9.8)	77.8 (13.2)	<0.001
Ease of intubation (1–10), mean (SD)	27.5 (7.4)	40.8 (18.6)	<0.001

Legend: MAC = Macintosh laryngoscope; FPS = first pass success rate; SD = standard deviation; POGO = percentage of glottis opening.

## Data Availability

The data that support the findings of this study are available on request from the corresponding author (L.S.).
